# Association between Psychological Stress and Neck Pain among College Students during the Coronavirus Disease of 2019 Pandemic: A Questionnaire-Based Cross-Sectional Study

**DOI:** 10.3390/healthcare9111526

**Published:** 2021-11-09

**Authors:** Amira Daher, Ofra Halperin

**Affiliations:** 1 Department of Physical Therapy, Safed Academic College, Safed 1320611, Israel; 2 Department of Nursing, Max Stern Academic College of Emek Yezreel, Emek Yezreel 19300, Israel; ofrah@yvc.ac.il

**Keywords:** neck pain, COVID-19, lockdown, psychosocial factors, musculoskeletal diseases, survey, lifestyle factors, Perceived Stress Scale

## Abstract

The coronavirus disease of 2019 (COVID-19) greatly affected people’s lifestyles. We used an online, cross-sectional survey during a COVID-19-related lockdown in Israel, with the aim of investigating the effects of such lockdowns on students’ self-perceived stress and neck pain (NP). College students (*N* = 295) completed questions on sociodemographic characteristics, the Neck Disability Index (NDI), the Perceived Stress Scale, the Visual Analogue Scale (VAS), and NP frequency (four-point scale). Logistic regression models were calculated with the NDI as the dependent variable. In total, 35.6% of students experienced at least moderate NP-related disability (NDI ≥ 15), more during than before the lockdown. NP increased gradually, from a lifetime mean of 1.80 to a lockdown mean of 3.07 (χ^2^ = 316.72; *p* < 0.001). Students’ self-perceived stress was moderate, and 59.3% reported experiencing study-related stress. Higher levels of self-perceived stress, study-related stress, sitting >3 h/day, and a higher VAS score were associated with a higher risk of an NDI ≥ 15 (Nagelkerke’s *R*^2^ = 0.513, *p* < 0.001). The transition from on-campus to online learning seems to have exacerbated students’ NP, which was correlated to their study stress and lifestyles. These findings advocate for the need to promote the physical and mental health of students via e.g., mental health services and occupational and ergonomic consulting services.

## 1. Introduction

The coronavirus disease of 2019 (COVID-19) pandemic brought enormous changes to people’s lifestyles, including to college students. This manifested in anxiety and depression [1], as well as negative effects on the musculoskeletal system [2]. The World Health Organization and public health authorities around the world acted to contain the COVID-19 outbreak [3]. Many countries, including Israel, implemented periods of lockdown to reduce the spread of the virus. Beginning with the first lockdown, which started on 15 March 2020, academic classes in Israel were required to shift from on-campus to online learning, which changed students’ habits and lifestyles [2,4,5].

In a recent meta-analysis, Barta et al. (2021) [6] presented evidence that mental health issues among college students is a leading public health concern, which was seemingly exacerbated during the COVID-19 pandemic. The pandemic and consequent lockdowns were reportedly associated with depressive symptomatology among both office workers and students [2,3]. Home confinement, physical inactivity, a lack of an academic schedule, and stress among students also reportedly led to symptoms of hormonal imbalances [2].

Lockdown is an effective way to prevent the transmission of the COVID-19 virus. Yet it may also have negative effects on students’ mental health [7,8] and their musculoskeletal system [2,7]. Neck pain (NP), one of the most commonly reported musculoskeletal disorders, is a major cause of illness, reduced educational attainment, and absence from university lessons; NP may thus place students’ career prospects in jeopardy [9]. In addition to the general factors that predispose people to experience NP, students spend long hours reading, writing, and using computers or tablets, making them a high-risk group for the development of NP [10].

Kim et al. [11] demonstrated a statistically significant positive correlation between psychological factors, such as a depressed mood and anxiety, and NP. Psychosocial stress can increase muscle activity, leading to a higher mechanical load and musculoskeletal pain [12,13].

Over the past decades, NP has become a prominent health problem, exerting a considerable socioeconomic impact on individuals, families, communities, and the healthcare system [2,9,10,11,14]. Most patients with disabling NP in one study experienced a decrease in pain intensity over 1 year; however, a quarter of them experienced no or minimal improvement [15].

Although many cross-sectional and epidemiological studies have been conducted to examine the prevalence of lower back pain in students [16,17,18], far fewer studies were focused on NP and its association with factors pertinent to students, such as excessive exposure to screens, a maladaptive ergonomic environment, and psychological stress [19,20]. Indeed, musculoskeletal pain in students manifests most often in the neck [19]. Yet, there is a lack of knowledge about the development of NP among students during the COVID-19 pandemic.

Staying at home increases the risk of leading a sedentary lifestyle, an excessive amount of screen time, and abnormal sleeping habits, all of which may result in a higher body mass index (BMI) [2,21]. During home confinement, undergraduate students reportedly experience chronic stress, headaches, and lower back pain [6,18]. Thus, we hypothesized that the prevalence of NP among students was higher during the COVID-19 pandemic than in the period prior to the pandemic. 

In this cross-sectional study, we designed an online survey with the aim of evaluating the prevalence of NP among college students in Israel during the third lockdown compared to what they recalled of that during the pre-pandemic period. We also aimed to assess the impact of psychological stress, sociodemographic factors, and lifestyle (such as physical activity and seated hours a day) on NP. Empirical research is needed to capture the effect of the pandemic on students’ mental and physical health and to support decision-making to manage these conditions. Based on previous studies, we hypothesized that, during the COVID-19 pandemic, students’ NP symptoms were exacerbated and correlated to psychological stress and various lifestyle factors [7,22].

## 2. Materials and Methods

### 2.1. Study Design and Ethics

A cross-sectional study was conducted in the form of an online, questionnaire-based survey posted at the Emek Yezreel College in January 2021; data were collected during the third COVID-19 lockdown in Israel, from 7 January to 8 February 2021. Students at our college participated in the study. The study had no exclusion criteria. The survey was circulated to students via email. Their informed consent was obtained for use of their data for this study. Participants recorded their responses on the Qualtrics survey platform. To prevent multiple responses from the same participants, only one response was allowed per item.

The survey was conducted in adherence to the Helsinki Declaration and approved by the Departmental Research Ethics Committee, Emek Yezreel College (Approval Number YVC EMEK 2021-17). The survey conformed to the recommendations of the STROBE Statement.

### 2.2. Measurements

#### 2.2.1. Sociodemographic, Clinical, and Behavioral Characteristics

Sociodemographic and clinical questionnaire: The questionnaire was based on a Hebrew questionnaire on lower back pain that previously achieved reliable results [17,18]. We modified this questionnaire for NP. The questions were related to sociodemographic variables such as gender, age, height, weight, and BMI (kg/m^2^); medications for NP taken in the last month (yes/no); marital status, having children (yes/no), and the number of children; religiosity (secular, partly religious, religious); the department of studies: health sciences, nursing, or social sciences; the degree being pursued (bachelor’s or master’s degree); and the year of studies (from first to fourth year).

Participants were also asked about their health habits: smoking status and engagement in physical activity during their leisure time (yes/no). Physical activity was measured as hours per week: 1 to 2 h/week, >2 to 3 h/week, or >3 to 4 h/week. Participants were also asked to report their average seated hours per day, as determined by the sample item: “How many hours a day do you spend in a sitting position?”. The four categories were as follows: <1 h/day, 1 to 3 h/day, >3 to 5 h/day, and >5 h/day.

#### 2.2.2. Measuring Tools 

Neck Disability Index (NDI) (Vernon and Mior, 1991) [23]: Participants answered 10 questions regarding the NDI, each rated on a five-point scale, ranging from 0 = “painless” to 5 = “worst pain imaginable.” The maximum possible score was 50. A higher score indicated a higher degree of neck disability, and the NDI yielded a value for Cronbach’s alpha of 0.87 in this study. The cut-off value of the NDI for detecting NP associated with disability was determined as 15, such that 0–14 indicated no disability, while a score of 15 and up pointed to disability [24].

Perceived Stress Scale (PSS) (Cohen et al, 1994) [25]: Participants answered questions about their feelings and thoughts over the past month on a four-point scale (1 = “never”; 4 = “often”). The questionnaire score is calculated by averaging the item scores. Cronbach’s alpha for this questionnaire was 0.89 in this study. The questionnaire includes eight positively worded items (items 4–10 and 13) and six negatively worded items (items 1–3, 11, 12, and 14). Higher scores indicate higher levels of perceived stress.

“Study—related stress”: Participating students answered one question regarding their “study-related stress” (“During the last month, how much stress have you felt related to your studies?) on a scale ranging from 1 (“none”) to 4 (“a lot”) [17,18]. Because of the distribution of this variable, the responses were dichotomized into “high” vs. “some.”

Pain intensity: The visual analogue scale (VAS) (Price et al, 1983) [26] is a 10-cm line, oriented vertically or horizontally, with one end representing “no pain” and the other end representing “pain as bad as it can be.” Patients are asked to mark the place on the line corresponding to their current pain intensity. The VAS is the most frequently used pain measure because it is simple to use and yields satisfactory validation results [26].

NP frequency: Participants were asked to mark the frequency with which they experienced NP at four time points: over their lifetime, during the last year, during the last six months, and currently (third lockdown in Israel), on a four-point scale ranging from 1 (never/seldom) to 4 (almost every day). NP was defined, in accordance with the Bone and Joint Decade 2000–2010 Task Force, as an ache or discomfort in the anatomical region of the neck, with or without radiation to the head, trunk, and upper limbs [27]. The posterior neck region is defined as the region “from the superior nuchal line to the spine of the scapula and the side region down to the superior border of the clavicle and the suprasternal notch” [28]. 

### 2.3. Statistical Analysis

The data were analyzed with IBM SPSS Statistics for Windows, version 27 (IBM Corp., Armonk, NY, USA). The background characteristics and study variables were described as means and standard deviations (SDs) for continuous variables, and as frequencies and percentages for categorical variables. Internal consistencies were calculated for the study variables by using Cronbach’s α. Simple logistic regression models with one independent variable were calculated for all background characteristics and study variables, with the NDI (<15 vs. ≥15) [24] as the dependent variable.

NP frequencies at the four time points were compared by using Friedman’s χ^2^ test and Wilcoxon’s signed-rank test. A multiple logistic regression model was used to assess the odds of having at least a moderate neck-related disability. The independent variables in this model were age, physical activity during leisure time, seated hours per day, study-related stress, PSS score, VAS score, and the use of medications for NP during the past month. None of the continuous variables deviated from a normal distribution (skewness = −0.27 to 1.49, standard error of the mean = 0.14). Correlations among the independent variables ranged from *r* = −0.19 to *r* = 0.44 (*p* < 0.001), and no collinearity was detected (maximum variance inflation factor = 1.41). The significance level was set at 0.05. As a result of the online data-collection protocol, there were no missing data.

### 2.4. Sample Size

Sample size was calculated with G*Power 3.1 [Heinreich-Heine-Universität, Düsseldorf, Germany] for a logistic regression with a minimum odds ratio (OR) of 1.5, α = 0.05, and a power of 0.80 [29]. Accordingly, the required sample size was 308. The actual sample size was 295, which resulted in a power of 0.80 for an OR = 1.6.

## 3. Results

### 3.1. Characteristics of Participants

A total of 295 college students participated in the study. Table 1 summarizes their demographic, academic, and behavioral characteristics during the pandemic. Most of the participants were female (84.7%) and aged in their twenties (mean = 27.7 years, SD = 8.32 years). The majority were single (67.5%) and had no children (73.9%). Almost all were studying for a bachelor’s degree (94.9%) and a majority were studying in the health sciences or nursing departments (76.6%). Most did not smoke (81.4%) and most female participants were not pregnant (95.6%) at the time of the study. In total, 45.8% of the participants engaged in some form of physical activity during leisure time. A majority (75.6%) spent ≥3 h/day seated, and approximately 50% spent more >5 h/day seated. Almost one-third of the participating students had taken medication for NP during the last month.

### 3.2. Prevalence of NP Symptoms and Differences in Sociodemographic Characteristics

According to the NDI, about a third of the participants (*n* = 105, 35.6%) experienced at least moderate neck-related disability (NDI > 15), while about two-thirds (*n* = 190, 64.4%) did not. A comparison of the demographic, background, and clinical characteristics according to the NDI cut-off value revealed no significant differences for demographic or background characteristics (Table 1). Interestingly, no significant differences were observed for several clinical characteristics: BMI, smoking status, physical activity, and weekly hours of physical activity. Seated hours were associated with the NDI, such that those who sat >3 h/day were at a significantly higher risk for at least a moderate level of neck-related disability than those who were seated for shorter amounts of time. Reporting at least moderate neck-related disability was related to higher odds of using medications for NP and experiencing higher levels of pain.

### 3.3. NP Intensity and Frequency at Four Time Points

**Figure 1:**
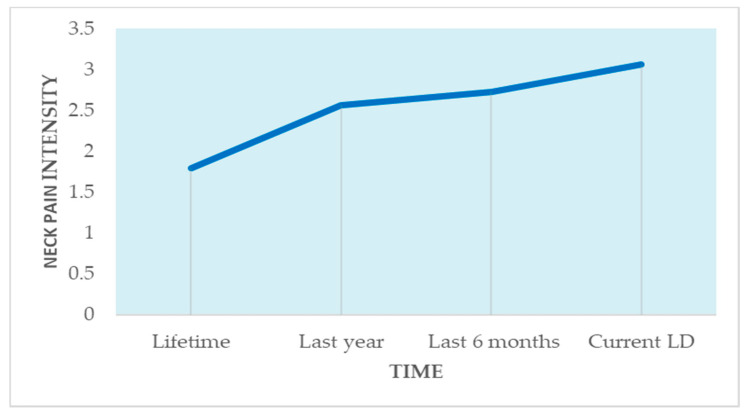
Neck pain at four time-points: lifetime, last year, last 6 months, and current, on a scale of 1–4. Abbreviation: LD: lockdown.

Reported NP increased gradually and significantly, from a lifetime mean of 1.80 (SD = 1.01), to a mean of 2.57 (SD = 1.13) during the last year, a mean of 2.73 (SD = 1.10) during the past six months, and a current mean of 3.07 (SD = 1.11) (χ^2^ [3 degrees of freedom] = 316.72, *p* < 0.001) (Figure 1). Current NP frequency was highly associated with the VAS score (*r* = 0.68, *p* < 0.001), as well as with the NDI (*r* = 0.63, *p* < 0.001).

### 3.4. Psychological Stress of College Students during the Lockdown

Participating students’ mean PSS score was moderate, at about 2.5 on a scale of 1–4 (Table 2). Reported stress was significantly associated with the NDI, such that a higher level of stress was associated with greater odds of experiencing at least a moderate level of neck-related disability.

### 3.5. Study-Related Stress

A majority (*n* = 175, 59.3%) of the students reported experiencing study-related stress. Study-related stress exhibited a significant association with the NDI, such that reporting the presence of study-related stress was associated with experiencing at least moderate neck-related disability. The two measures of stress were positively correlated (*r* = 0.42, *p* < 0.001).

### 3.6. Factors Associated with Moderate NP-Related Disability

A logistic regression model was used to assess the odds of reporting at least a moderate level of neck-related disability (NDI > 15) (Table 3). Other background variables were not entered into the model because they were not related to the NDI (Table 1). The logistic model was significant (χ^2^ [degrees of freedom = 6] = 118.38, *p* < 0.001), explaining about 46% of the variance in NDI (Nagelkerke’s *R*^2^ = 0.457). Sitting for >3 h/day, experiencing study-related stress, a higher PSS score, and a higher VAS score, were associated with a greater risk of experiencing at least a moderate level of neck-related disability.

## 4. Discussion

We set out to investigate how the COVID-19 pandemic-related lockdowns affected students’ self-perceived stress and musculoskeletal symptoms of NP, compared to the pre-pandemic period. The findings revealed that, during the third lockdown in Israel (7 January to 8 February 2021), students’ symptoms of NP seem to have been exacerbated and correlated to psychological stress and lifestyle habits. Although lockdown may be an effective measure to prevent the spread of the COVID-19 virus, previous studies indicated that the shift in academic studies from on-campus to online classes had negative effects on students’ mental [1,2,6,31] and musculoskeletal health [2,7]. 

Our results revealed that students were adversely affected by the lockdown in terms of NP frequency, which increased from lifetime frequency to that of the last year, last six months, and the third lockdown in Israel. It is worth noting that this was a cross-sectional study, in which the assessment was performed at only one time point during the third lockdown, using participant recall to assess outcomes for different periods of time. To the best of our knowledge, the current study is the first in which NP frequency was assessed at different time points among college students, particularly comparing that during the COVID-19 lockdown to that before the lockdown. This result is in agreement with the general tendency that was reported recently with regard to the negative effect of COVID-19 on the prevalence and severity of pain in musculoskeletal disorders [2,7,32,33,34].

We observed that the prevalence of moderate-to-severe NP among college students during the COVID-19 pandemic period (35.6%) fell within the range (22.3% to 75.1%) that has previously been reported among college students worldwide, even though these previous studies were conducted before the COVID-19 pandemic [32,35,36,37]. According to our findings, the frequency of NP during the lockdown was higher than during the COVID-19 pandemic in general (the last year and the last 6 months). NP reported during the lockdown was highly associated with the VAS score and the NDI. Fallon et al. [7] discovered that pain symptoms had increased in participants with chronic pain during the lockdown compared to the period before the lockdown. The increased frequency of NP during lockdown compared to previous periods (the last year and the last 6 months) may be explained by the fact that the study was conducted during exceptional circumstances (the COVID-19 pandemic), with heightened stress due to coronaphobia [8]. These findings highlight that the lockdown was the most difficult pandemic period for participants, with an increase in both severity and frequency of NP compared with previous periods.

Our results contradict those of Majumdar et al. [2] and Leirós-Rodríguez et al. [38], in which the pandemic did not have an adverse effect on NP in students. The results of other studies [5,39] were correlated with ours in terms of the adverse effect of the pandemic on NP among college students. We hypothesize that this discrepancy between the studies is related to the difference in the timing of the studies. The former studies [2,38] were conducted at the beginning of the pandemic, during the first lockdown (March–June 2020); however, the recent studies [5,39], including ours, were conducted about a year after the virus outbreak. Our study was conducted during the third lockdown in Israel, by which time cumulative fatigue and stress may have led to an increase in NP among students.

Based on previous studies, we hypothesized that, during the COVID-19 pandemic, students’ symptoms of NP were exacerbated and correlated to psychological stress [7,22]. The participants in our study reported a moderate level of stress during the lockdown. Moreover, about 60% of them reported experiencing study-related stress. Furthermore, we found an association between higher levels of self-reported stress and higher odds for experiencing at least a moderate degree of NP-related disability. NP is reportedly associated with psychosocial and mental stress [2], and stress is reportedly a strong predictor of neck/shoulder pain [40]. Similarly, a high degree of mental stress is considered a risk factor for musculoskeletal disorders of the neck [41]. 

The large percentage of students experiencing psychological stress is in agreement with the findings of previous studies [22,31,42,43]. A meta-analysis conducted by Chang et al. [1] indicated that college students worldwide were prone to feeling anxious and depressed during the COVID-19 pandemic. According to Marelli et al. [31], the lockdown in Italy had a statistically significant impact both on sleep and on psycho-emotional well-being, more so among students than among administrative workers, and more so among women than among men. Similarly, Li et al. [22] discovered that 16.3% of students at Wuhan universities and colleges had posttraumatic stress disorder four months after the COVID-19 pandemic.

The results of the current study indicate that sitting for more than three hours a day is associated with a greater risk of experiencing at least a moderate level of NP-related disability. The lockdown increased people’s reliance on electronic devices to connect with others, which in turn resulted in more time spent sitting in front of a screen. In a recent systematic review, it was determined that working in a sustained awkward position is a risk factor for the development of NP [11].

One surprising result of the current study is that engagement in physical activity during the lockdown was not associated with NP-related disability. This finding is inconsistent with those of previous studies, in which physical activity reduced NP [44,45] and served as a protective factor against NP [11]. However, most risk factors for NP were psychosocial rather than physical characteristics [11]. We believe that the stress experienced by students during the pandemic period was the dominant factor in NP, irrespective of engagement in physical activity.

Our results also indicate that older age and smoking among students were not related to NP and related disability during the lockdown period. These results are consistent with those of a recent systematic review [11].

We found no association between gender and the prevalence of NP, in contrast with other studies [45,46,47] in which chronic musculoskeletal pain was more common in women than in men. We believe that this result may be explained by the fact that most of participants in our study were nurses (and 85% of participants were females). This was entirely driven by increased levels of uptake among women, despite the fact that our recruitment process was aimed at both genders.

### Limitations

This was a cross-sectional retrospective study of a small number of participants (*n* = 295). Several potential sources of bias may be identified. First, potential participants may have chosen not to respond to the online questionnaire. Their characteristics compared to those of students who completed the questionnaire are unknown, constituting a limitation of the study. Second, social desirability may have biased the respondents’ answers. For that reason, the data were collected anonymously. Third, the cross-sectional, self-reported nature of the study constituted another limitation, as reporting may have been affected by current emotions or cognitions. As participants were questioned about their NP frequency of four time points, the latest experience may have been more vivid or meaningful to their minds than the earlier experiences. Finally, a larger sample size is needed for the conclusions of the current study to be generalizable.

## 5. Conclusions

The long-lasting COVID-19 pandemic and associated lockdown restrictions have had negative impacts on academic institutions. Our study indicated that the transition from on-campus to online learning during lockdown has a negative effect on students’ psychological stress as well as on musculoskeletal symptoms of NP. NP seems to have been exacerbated in students during the pandemic and was correlated to study-related stress and lifestyle factors (time seated per day).

This study has several implications. As it was an empirical study conducted throughout the pandemic lockdown period, it helps to clarify the implications of the pandemic on students. The association between NP and psychological stress factors highlight the need to develop interventions and preventive strategies to promote the physical and mental health of students. Mental health services and occupational and ergonomic consulting may provide advice for a healthy lifestyle, especially among students who are seated for prolonged amounts of time.

## Figures and Tables

**Table 1:** Demographic, background, and health-related characteristics, for the total sample and by NDI (*N* = 295).

Variable		Total	NDI up to 14 (*n* = 190)	NDI 15 and Higher (*n* = 105)	OR (95% CI)	*p*
Gender, *n* (%)	Female	250 (84.7)	158 (83.2)	92 (87.6)	1.43 (0.72, 2.87)	0.301
	Male	45 (15.3)	32 (16.8)	13 (12.4)	1.00 (reference)	
Family status, *n* (%)	Single	199 (67.5)	128 (67.4)	71 (67.6)	0.55 (0.13, 2.28)	0.677
	Married	88 (29.8)	58 (30.5)	30 (28.6)	0.52 (0.12, 2.21)	
	Divorced/ widowed	8 (2.7)	4 (2.1)	4 (3.8)	1.00 (reference)	
Children, *n* (%)	Yes	77 (26.1)	50 (26.3)	27 (25.7)	0.97 (0.56, 1.67)	0.910
	No	218 (73.9)	140 (73.7)	78 (74.3)	1.00 (reference)	
Religiosity, *n* (%) (*n* = 272)	Secular	132 (48.5)	86 (48.6)	46 (48.4)	1.32 (0.66, 2.65)	0.497
	Partly religious	88 (32.4)	54 (30.5)	34 (35.8)	1.55 (0.74, 3.25)	
	Religious	52 (19.1)	37 (20.9)	15 (15.8)	1.00 (reference)	
Department, *n* (%)	Health, Nursing	226 (76.6)	148 (77.9)	78 (74.3)	0.82 (0.47, 1.43)	0.485
	Social Sciences	69 (23.4)	42 (22.1)	27 (25.7)	1.00 (reference)	
Degree, *n* (%)	BA	280 (94.9)	180 (94.7)	100 (95.2)	--	--
	MA	15 (5.1)	10 (5.3)	5 (4.8)	--	
Year of studies, *n* (%)	First	132 (44.7)	91 (47.9)	41 (39.0)	1.69 (0.53, 5.40)	0.144
	Second	90 (30.5)	52 (27.4)	38 (36.2)	2.74 (0.84, 8.91)	
	Third	54 (18.3)	32 (16.8)	22 (21.0)	2.58 (0.75, 8.81)	
	Fourth	19 (6.4)	15 (7.9)	4 (3.8)	1.00 (reference)	
Age, years, M (SD)	19–57	27.73 (8.32)	27.88 (8.18)	27.46 (8.59)	0.74 (0.29, 1.87)	0.523
Number of children, *n* (%), M (SD)	1–4	2.40 (1.02)	2.36 (1.00)	2.48 (1.05)	1.13 (0.71, 1.79)	0.614
BMI, kg/m^2^, M (SD)	15.6–41.2	24.54 (5.02)	24.45 (5.02)	24.69 (5.04)	1.01 (0.96, 1.06)	0.693
Smoke, *n* (%)	Yes	55 (18.6)	40 (21.1)	15 (14.3)	0.62 (0.33, 1.20)	0.146
	No	240 (81.4)	150 (78.9)	90 (85.7)	1.00 (reference)	
Pregnant (female), *n* (%) (*n* = 250)	Yes	11 (4.4)	8 (5.0)	3 (2.26)	--	--
	No	239 (95.6)	150 (95.0)	89 (96.7)	--	
Physical activity, *n* (%)	Yes	135 (45.8)	89 (46.8)	46 (43.8)	0.88 (0.55, 1.43)	0.617
	No	160 (54.2)	101 (53.2)	59 (56.2)	1.00 (reference)	
Physical activity- hours per week, *n* (%) (*n* = 135)	1–2 h	61 (45.2)	38 (42.7)	23 (50.0)	1.40 (0.66, 2.74)	0.420
	>2 to 3 h	39 (28.9)	28 (31.5)	11 (23.9)	1.00 (reference)	
	>3 to 4 h	35 (25.9)	23 (25.8)	12 (26.1)		
Seated hours per day, *n* (%)	<1 h	19 (6.4)	17 (8.9)	2 (1.9)	1.00 (reference)	<0.001
	1–3 h	53 (18.0)	45 (23.7)	8 (7.6)		
	>3 to 5 h	74 (25.1)	44 (23.2)	30 (28.6)	4.60 (2.24, 9.44)	
	>5 h	149 (50.5)	84 (44.2)	65 (61.9)		
Medications for neck pain, last month, *n* (%)	Yes	89 (30.2)	30 (15.8)	59 (56.2)	6.84 (3.95, 11.84)	<0.001
	No	206 (69.8)	160 (84.2)	46 (43.8)	1.00 (reference)	
Current pain, VAS score, M (SD)	0–10	4.85 (2.56)	3.99 (2.44)	6.41 (1.95)	1.63 (1.42, 1.88)	<0.001
VAS score, *n* (%)	0–2	49 (16.6)	47 (24.7)	2 (1.9)	1.00 (reference)	<0.001
	3–6	157 (53.2)	114 (60.0)	43 (41.0)	8.86 (2.06, 38.09)	
	7–8	73 (24.7)	26 (13.7)	47 (44.8)	42.48 (9.54, 189.24)	
	9–10	16 (5.4)	3 (1.6)	13 (12.4)	101.83 (15.36, 675.20)	

Abbreviations: NDI—Neck Disability Index; VAS—Visual Analogue Scale; OR—odds ratio; CI—confidence interval; M—mean; SD—standard deviation; BMI—body mass index. Note: VAS was categorized according to the recommendation of Reich et al. (2017) [30].

**Table 2:** Distribution of stress for the total sample and by NDI (*N* = 295).

**Variable**	**Range**	**Total**	**NDI ≤ 14** **(*n* = 190)**	**NDI ≥ 15** **(*n* = 105)**	**OR (95% CI)**	***p***
PSS, *M* (SD)	1–4	2.49 (0.52)	2.34 (0.47)	2.76 (0.50)	6.41 (3.58, 11.48)	>0.001
“Study-related stress” ^1^ *M* (SD)	0–1	0.59 (0.49)	0.47 (0.50)	0.82 (0.39)	5.14 (2.90, 9.11)	>0.001

^1^ Dichotomized into “high” vs. “some.” Abbreviation: PSS—Perceived Stress Scale.

**Table 3:** Logistic regression model for NDI (≥15) with background variables, physical habits, stress, and pain, (*N* = 295).

**Variable**	***B***	***SeB***	***OR* (95% *CI*)**	***p***
Age	0.03	0.02	1.03 (0.99, 1.07)	0.085
Physical activity (yes)	0.29	0.32	1.34 (0.72, 2.49)	0.356
Hours seated per day (>3 h)	1.40	0.43	4.07 (1.74, 9.50)	0.001
Study-related stress (high)	0.78	0.36	2.18 (1.08, 4.40)	0.030
PSS	1.37	0.37	3.95 (1.91, 8.17)	<0.001
VAS	0.44	0.08	1.55 (1.33, 1.81)	<0.001

Abbreviations: SeB—Standard Error of B.

## Data Availability

The datasets used and/or analyzed during the current study are available from the corresponding author on reasonable request.
